# Access to general practice: why policy must be rural-proofed

**DOI:** 10.3399/BJGP.2026.0187

**Published:** 2026-06-25

**Authors:** Helen Leach, Daniel James, Sonum Bathia, Emma Watts

**Affiliations:** 1 Unit of Academic Primary Care, Warwick Medical School, University of Warwick, Coventry, UK; 2 Woolpit Health Centre, Woolpit, Bury St Edmunds, UK; 3 Shere Surgery and Dispensary, Shere, UK

Access 8to general practice has become a defining focus of NHS policy and political debate in recent years. Reforms to the GP contract include commitments for same-day appointments for patients with urgent needs alongside an expansion of digital access, aligning with the *Fit for the Future: 10 Year Health Plan for England* and its agenda for digital transformation and neighbourhood-based models of community care.^1^

Yet these ambitions risk obscuring a more fundamental challenge. Access is not simply a matter of speed but an alignment between services, patient need, practice capacity, and local context. Viewed through the lens of geography, the current policy priorities come under strain.

Rural-proofing is the systematic analysis of policy to ensure that design and implementation account for the realities of rural populations, including infrastructure, workforce, and access constraints, and offers a practical corrective to urban-centric policies. Without this lens being integrated routinely, reforms risk systematically disadvantaging the communities they are intended to serve.

*“Rural-proofing is the systematic analysis of policy to ensure that design and implementation account for the realities of rural populations ... ”*

## Understanding access

In rural general practice, access encompasses multiple dimensions: timely contact, geographical reach, adaptability to patient circumstances, and integration with community resources. Rural communities make up over a quarter of the population in Northern Ireland, Wales, and Scotland, and around 17% in England.^2^ Across all four nations, rural areas are home to increasingly older populations driven by in-migration, colloquially termed the ‘escape to the country’ effect.^2^ Rural general practice must therefore provide high-quality, accessible care to older, often multimorbid, complex populations dispersed across wide geographic areas.

Continuity of care also remains core in rural communities. Patients often rely on long-standing relationships with clinicians who not only understand their medical histories, but also the practical realities of their lives, including transport limitations, social networks, and caring responsibilities. Continuity, associated with improved outcomes, reduced hospital admissions, and greater patient satisfaction,^3^ is declining across general practice, but remains higher within rural practices.^4^ Policies prioritising rapid transactional access risk undervaluing the relational nature of general practice and, without rural-proofing, overlook the important role of continuity.

Distance and sparse populations pose fundamental challenges, leaving patients reliant on personal vehicles, informal support from friends and family, or expensive taxis. Metrics such as same-day access, when applied without rural-proofing, risk misrepresenting access in communities where geography and transport fundamentally shape care use.

The introduction of neighbourhood health centres is likely to intersect with rurality, deprivation, and geographical dispersion.^5^ The postcode lottery in access, depending on how local systems configure neighbourhood boundaries and services, is real and warrants explicit consideration in implementation guidance.

## Funding and workforce

Resource allocation shapes rural practice capacity. Deprivation indices have not consistently captured rural disadvantage, reinforcing images of rural idyll that often do not match lived experiences.^6^ This has led to reduced funding for many rural practices serving populations with high care needs and soaring delivery costs.^7^ While the latest Index of Multiple Deprivation has begun to address the relationship between rurality and deprivation,^8^ how this will translate into meaningful change in funding allocation remains to be seen.

Workforce availability compounds these pressures. Recruitment and retention are more difficult in rural areas, resulting in smaller practice teams and higher per-clinician workloads.^9,10^ Rural GPs often work with greater clinical autonomy, managing complex presentations with limited access to secondary care, a reality at odds with perceptions of ‘sleepy, countryside’ general practice. Financial incentives have been used to address recruitment. The Targeted Enhanced Recruitment Scheme offered a golden handshake for GP registrars entering under-filled programmes, including rural areas, and was associated with increased odds of recruitment.^11^ Although no long-term impact was evaluated, the effectiveness of financial incentives on recruitment and retention is mixed.^12^ Without targeted workforce strategies that account for the professional and personal dimensions of working in rural areas, reforms that focus solely on appointment metrics will not necessarily improve the care of rural patients amid workforce pressures.

## Digital transformation

Digital and remote options, including online consultations, remote triage, or online appointment booking, offer opportunities to improve efficiency and reduce unnecessary travel.^13^ But remote consulting is not a panacea for rural populations; despite being the population theoretically benefitting the most from reduced travel, they are the same communities that have the least infrastructure to support remote routes. Poor broadband connectivity, patchy mobile signal, and widespread power cuts are structural barriers that fundamentally limit the real-world value of remote models. The advancement of the digital transformation policy, without prospectively assessing the impact on rural communities, represents a failure of rural-proofing.

Moreover, the intersection of rurality with groups most at risk of digital exclusion, such as older or more deprived populations, requires more exploration. Even where infrastructure exists, many patients, particularly those of older age, require additional support to navigate digital services effectively, a role lacking in current policy.^14^ For rural general practice, a model based on patient choice that enables digital access when it works, while protecting in-person access when it doesn’t, is likely to be more equitable than one that assumes digital engagement as a default.

*“Rural-proofing should not be an afterthought but a core design principle if policy is to achieve equitable outcomes across diverse geographies.”*

## The case for rural-proofing

Standard policy too often fails to reflect the rural general practice context. Rural-proofing offers a corrective: a systematic process testing national policy against rural needs, geographies, and services, evaluating what will work when applied outside urban settings where most policy thinking originates.

Rural-proofing is not a new concept. Multiple governments have purported to consider rural impact as part of policy development; however, in practice, its application has been inconsistent. There has been a conflation of rural-proofing with ‘levelling up’, with urban experiences used as benchmarks rather than recognition of difference.^15^ The Rural Proofing for Health Toolkit and the work of the National Centre for Rural Health and Care offer structured approaches but remain underused within mainstream NHS policy development.^16^ Rural-proofing, when done properly, requires prospective assessment, not retrospective recognition when policy has not worked for rural communities.

The absence of rural-proofing can be seen across policy and practice. A policy mandating same-day access may appear desirable, but reflects urban service design and fails to account for patients who cannot physically reach services due to distance or transport. Services designed with rurality in mind, as detailed throughout the Rural Proofing for Health Toolkit as case-studies, are more often flexible and person-responsive, able to reflect the heterogeneity across rural communities.^16^ This has included digital methods, for example, but infrastructure and patient needs have been considered rather than assumed.

Rural-proofing also requires an evidence base that has historically been lacking. Rural populations are frequently aggregated within larger urban datasets, hiding their specific experiences. Dedicated research into rural primary care is essential if policy is to be genuinely fit for purpose, and major funders should explicitly recognise rurality as a disadvantaged population to target.

## Parting thoughts

Equitable access to general practice does not emerge from uniform targets applied regardless of context, but from services designed to fit the realities of patients and clinicians in places they live and work.

Rural general practice serves communities that are older, more dispersed, and often more complex than national policy frameworks tend to assume. Rural-proofing should not be an afterthought but a core design principle if policy is to achieve equitable outcomes across diverse geographies.
